# Sporulation Genes Associated with Sporulation Efficiency in Natural Isolates of Yeast

**DOI:** 10.1371/journal.pone.0069765

**Published:** 2013-07-17

**Authors:** Parul Tomar, Aatish Bhatia, Shweta Ramdas, Liyang Diao, Gyan Bhanot, Himanshu Sinha

**Affiliations:** 1 Department of Biological Sciences, Tata Institute of Fundamental Research, Mumbai, India; 2 Department of Physics and Astronomy, Rutgers University, Piscataway, New Jersey, United States of America; 3 BioMaPS Institute for Quantitative Biology, Busch Campus, Rutgers University, Piscataway, New Jersey, United States of America; 4 Department of Molecular Biology and Biochemistry, Rutgers University, Piscataway, New Jersey, United States of America; 5 Cancer Institute of New Jersey, New Brunswick, New Jersey, United States of America; 6 Simons Center for Systems Biology, Institute for Advanced Study, Princeton, New Jersey, United States of America; University of Cambridge, United Kingdom

## Abstract

Yeast sporulation efficiency is a quantitative trait and is known to vary among experimental populations and natural isolates. Some studies have uncovered the genetic basis of this variation and have identified the role of sporulation genes (*IME1*, *RME1*) and sporulation-associated genes (*FKH2*, *PMS1*, *RAS2*, *RSF1*, *SWS2*), as well as non-sporulation pathway genes (*MKT1*, *TAO3*) in maintaining this variation. However, these studies have been done mostly in experimental populations. Sporulation is a response to nutrient deprivation. Unlike laboratory strains, natural isolates have likely undergone multiple selections for quick adaptation to varying nutrient conditions. As a result, sporulation efficiency in natural isolates may have different genetic factors contributing to phenotypic variation. Using *Saccharomyces cerevisiae* strains in the genetically and environmentally diverse SGRP collection, we have identified genetic loci associated with sporulation efficiency variation in a set of sporulation and sporulation-associated genes. Using two independent methods for association mapping and correcting for population structure biases, our analysis identified two linked clusters containing 4 non-synonymous mutations in genes – *HOS4*, *MCK1*, *SET3*, and *SPO74*. Five regulatory polymorphisms in five genes such as *MLS1* and *CDC10* were also identified as putative candidates. Our results provide candidate genes contributing to phenotypic variation in the sporulation efficiency of natural isolates of yeast.

## Introduction

Sporulation is a response to nutrient deprivation in which yeast exits mitotic cell cycle and enters into meiosis, leading to spore formation [1]. About 400 genes have been shown to modulate sporulation [2], [3] and more than 1,000 genes are known to change expression during sporulation [4], [5]. Sporulation efficiency, defined as the fraction of cells that sporulate in a culture, varies among strains and has been identified as a quantitative trait that is modulated by at least 9 genes [6]–[8]. However, many of these studies have been performed using laboratory strains [6], [7], which face distinct selective pressures as compared to wild type strains.

The lack of information about traits in natural populations has limited our understanding of the potential effects of evolution, selection pressure, life history and environment on trait variation and its mechanism of action. Sporulation is triggered as a response to nutrient deprivation. As natural isolates face strong selection pressure to adapt to nutrient changes in their environment, it is reasonable that mechanisms causing variation in sporulation efficiency in natural isolates may be very different from those operating in laboratory strains.

Several previous studies have shown variation of sporulation efficiency among natural isolates of yeast, such as clinical, oak and wine strains [8]–[11]. To understand this variation among a larger set of natural isolates and to identify some of the genetic factors contributing to this phenotype, we measured sporulation efficiency of strains in the SGRP collection [12]. While a previous study has shown large variation in sporulation efficiency in SGRP strains [11], our goal was to examine whether the genes that have been implicated in sporulation to date [1], [3] also contribute to sporulation efficiency variation in these SGRP strains. This would help us understand how sporulation efficiency variation is modulated in natural isolates from diverse environmental niches.

To identify loci associated with sporulation efficiency in SGRP collection, we used two methods of association mapping in a set of 397 sporulation and sporulation-associated genes (Table S1). After correcting for population structure, indicated in SGRP strains, we identified two significant clusters of SNPs in strong linkage disequilibrium that were strongly associated with high sporulation efficiency. The SNPs were found in *HOS4*, *MCK1*, *SET3*, *SPO74* and other candidate genes.

## Materials and Methods

### Yeast Strains and Culture Conditions

Yeast strains were obtained from the Saccharomyces Genome Resequencing Project (SGRP) [12]. All strains were grown under standard media and growth conditions. To measure sporulation efficiency, strains were first grown in YPD (yeast extract, peptone and dextrose) from a starting optical density (OD) at 600 nm of 0.2 to final OD of 1.0. Their cell cycle was then synchronized by growing them in YPA (yeast extract, peptone and acetate) from a starting OD of 0.2 to final OD of 1.0 at 30°C, shaking at 250rpm [13]. Approximately 1×10^7^ cells from this synchronized culture were then incubated in liquid sporulation medium (1% potassium acetate supplemented with amino acid mixture) at 30°C for the duration of experiment.

### Estimation of Sporulation Efficiency

For each strain, three biological replicates were used and approximately 1,000 cells were counted per replicate per strain. Sporulation efficiency was measured as the ratio of tetrads and dyads produced by a strain, to the number of cells (expressed as a percentage). For each strain, sporulation efficiency was measured every two days until saturation was reached for three consecutive readings (Table 1, Table S3).

**Table 1:** Sporulation efficiency measurement of SGRP strains.

Strains	Mean sporulation efficiency (%)^a^	Sporulation efficiency (from Cubillos *et al*. [11])^b^
273614N	NS	+++
322134S	NS	NA
378604X	NS	NA
BC187	61.3±0.9	++
DBVPG1106	22.4±1.2	+++
DBVPG1373	NA	+
DBVPG1788	40.4±0.9	NA
DBVPG1853	NS	+
DBVPG6040	67.4±1.2	+
DBVPG6044	6.0±0.9	++
DBVPG6765	NS	+++
K11	19.9±2.4	–
L-1374	76.6±1.4	+++
L-1528	70.2±1.0	+++
NCYC110	NS	+++
NCYC361	NA	–
S288c	NS	NA
SK1	92.4±1.8	+++
UWOPS03–461.4	86.8±1.2	+++
UWOPS05–217.3	88.5±1.2	+++
UWOPS05–227.2	85.2±1.8	+++
UWOPS83–787.3	98.6±0.4	+++
UWOPS87–2421	89.9±1.0	+++
W303	NA	NA
Y12	54.0±1.5	+
Y55	73.7±1.7	+++
Y9	22.1±2.8	–
YIIc17_E5	NS	++
YJM975	48.2±2.2	+++
YJM978	40.2±1.0	+++
YJM981	NS	+++
YPS128	99.0±0.6	+++
YPS606	97.9±0.5	+++
YS2	NS	–
YS4	NS	–
YS9	NA	NA

(a) Mean (with standard deviation) sporulation efficiency of each strain at saturation, *i.e.* when sporulation efficiency did not vary for three consecutive time points. (b) Sporulation efficiency as reported by Cubillos *et al.* [11]. The scale indicates: (+++) high, (++) medium, (+) low sporulation efficiency, (−) none, (NA) not applicable (either the strain was haploid or did not grow in YPA), (NS) did not sporulate and zero sporulation efficiency.

### Sequence Data

The sequence and SNP data for all strains was obtained from the SGRP project (http://www.sanger.ac.uk/research/projects/genomeinformatics/sgrp.html; downloaded in February 2012). Sequence alignments using the *Saccharomyces cerevisiae* genome as reference was done for each gene being analyzed, starting from 500 base pairs upstream of the gene. Alignment was done using the SGRP tool ‘*alicat.pl*’ (available for download at the SGRP database). Variant loci were identified and were analyzed for association with the phenotype.

### LOD Score Analysis

The data consisted of 42,003 SNPs with phenotype data for 32 strains. These SNPs were filtered to include only biallelic SNPs with no missing data and with minor allele frequency ≥2/32, leaving 10,481 SNPs. For each SNP, the LOD score [14] was calculated, which is the log (base 10) of the ratio of the likelihood of the data given the hypothesis that there is a QTL to the likelihood of the data given the hypothesis that there is no QTL. A LOD score of 3.0 implies that the likelihood that there is a QTL (*i.e*. the data are drawn from a distribution where the two genotypes have different phenotypic means) is 1,000 times greater than the likelihood that there is no QTL (*i.e.* the data are drawn from a distribution where the two genotypes have the same phenotypic mean).

Let *q_1_* and *q_2_* be the fraction of strains having allele 1 and 2, respectively, and *x* be the total number of strains. Let *v_1_* and *v_2_* be the phenotype variances of strains with alleles 1 and 2, and *v* be the overall phenotype variance. Then, for each SNP, the LOD score is given by
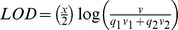



Permutation tests of up to 10^6^ permutations were done to assign an empirical p-value to each SNP. This test approximates the probability of observing a LOD score greater than or equal to a certain value, assuming the null hypothesis that there is no QTL at this SNP. The last step in this analysis was to correct for making multiple comparisons. To do this, we first grouped the 10,481 SNPs in our filtered data into clusters containing SNPs that were in perfect linkage disequilibrium. We then multiplied the permutation test p-value by the number of such clusters (*i.e.* we applied Bonferroni correction with n = 1,709). This left us with 2 clusters of SNPs with p-value <0.03 (corrected for multiple testing).

### Binomial Analysis

As a check on the LOD analysis, we also performed a binomial test on the data. The data consisted of 42,003 variant loci in genes potentially associated with sporulation (Table S1) and a measured sporulation efficiency value for 32 strains. After retaining only bi-allelic SNPs with no missing data and restricting to loci with minor allele frequency (MAF) >5/32 (∼0.16), 4,664 SNPs remained. The strains were stratified into 3 sets, broadly based on the sporulation efficiency classification used by Cubillos *et al*. [11], ranging from 0/1, 2 and 3. Set S_1_ contained 15 poor sporulation efficiency strains, with sporulation efficiency from 0% to 24%; set S_2_ contained 8 intermediate efficiency strains with sporulation efficiency from 25% to 74%, and S_3_ contained 9 high sporulation efficiency strains, with sporulation efficiency from 75% to 100%. Thus, the a-priori probabilities for a strain chosen at random to belong to set S_1_, S_2_, and S_3_ were 0.47, 0.25 and 0.28 respectively.

For each allele, a binomial test was applied to determine whether an allele at a SNP was significantly associated with set S_1_ (low sporulation efficiency) or with set S_3_ (high sporulation efficiency).

Let *n* be the number of samples with the major alleles and *k* the number of major alleles in class S_1_. Also, let *p* to be the a-priori probability for an allele to occur in class S_1_ (0.47). If there is no association between the major allele and low sporulation efficiency, the probability *P* of obtaining *k* or more major alleles in class S_1_ is given by:
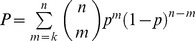



This is the p-value, or the probability of obtaining an association as extreme as the one seen in the data by chance, when in fact, the null hypothesis is true: *i.e.* when there is no association between the allele and sporulation efficiency.

For the 4,664 SNPs that remained after filtering, the p-value was computed as described above to test for the association of both minor and major alleles with high or low sporulation efficiency (4 comparisons per SNP). We used a significance threshold of p<0.05. For our final results, we retained only those SNPs identified as statistically significant by the LOD score analysis and by the binomial test, as being associated with the sporulation phenotype (Table 2, Table S4). Table S2 lists the LOD score, binomial test p-values, genotypes and mean phenotypes for the 69 SNPs that were identified with a LOD score >2.5.

**Table 2:** Clusters of SNPs with genome-wide significant LOD scores (Bonferroni corrected p-value <0.03).

SNPs in Linked Cluster	LOD score	Bonferroni Corrected p-value (n = 1,709)	Sporulation Efficiency of Minor Allele	Sporulation Efficiency of Major Allele
*HOS4∶1038,* *HOS4∶1206,* *MLS1:-21,* *SPR6:-434,* *TEP1∶219*	4.47	0.004	92.27	28.95
*CCR4∶2016,* *CDC10:-126,* *CIS1∶174,* *DOA1∶1152,* *EMI5:-10,* *GIP1∶213,* *HOS4∶1384,* *HPR1∶1137,* *HPR1∶1239,* *HPR1∶1293,* *MAF1∶761,* *MCK1∶1112,* *MPC54∶678,* *PEP12∶201,* *PEP12∶294,* *RAS2∶924,* *RME1∶63,* *SEF1∶1254,* *SET3∶1783,* *SHC1∶213,* *SPO74∶16,* *SPO75∶1842,* *SPR6∶519,* *SPR6∶426,* *SSN8:-484,* *VID28∶1410*	3.50	0.026	92.67	31.38

## Results

### Sporulation Efficiency Variation in SGRP Collection Strains

To uncover the genetic basis of variation in natural isolates, we used the SGRP collection, which consists of 36 sequenced, genetically diverse and highly polymorphic *S. cerevisiae* strains. We measured the sporulation efficiency of these strains and found extensive variation, ranging from strains that did not sporulate (322134S, 378604X, 273614N, YIIc17_E5), to ones that showed low 1–25% (DBVPG6044, K11, DBVPG1106, Y9), intermediate 25–49% (DBVPG1788, YJM975, YJM978), high 50–74% (Y12, Y55, BC187, DBVPG6040, L-1528), and very high 75–100% (L-1374, UWOPS05–227.2, SK1, YPS606, YPS128) sporulation efficiency (Table 1 and Table S3). We found that approximately one third (11 out of 32) of the strains failed to sporulate and their sporulation efficiency was set to zero in the association analysis. The inability of a number of natural isolates to sporulate may simply be because the conditions (temperature, media, aeration, *etc*. [15]) used to sporulate them in the lab might not be conducive to induce sporulation in these natural strains. Alternatively, it might be that these strains have inherently low sporulation efficiency and have developed alternate mechanisms to cope with nutrient deprivation, *e.g.* pseudo-hyphae as in case of YJM981 and 322134S.

**Figure 1:**
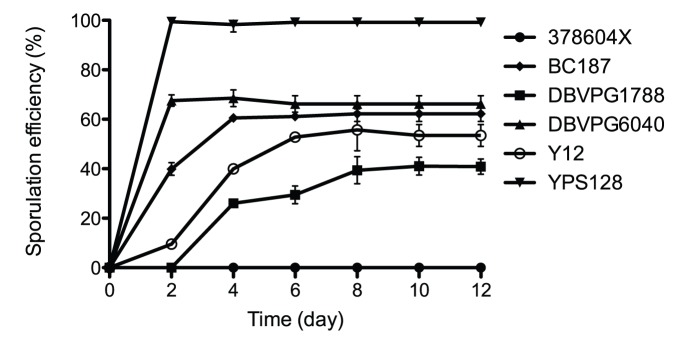
Kinetics of sporulation efficiency measurements of representative *S. cerevisiae* SGRP strains. Sporulation efficiency of each strain was measured till saturation, *i.e.* when sporulation efficiency did not vary for three consecutive time points. The data is plotted as mean and standard deviation of 3 independent biological replicates.

We repeatedly measured the sporulation efficiency of each strain at intervals of 48 h, till the efficiency saturated, *i.e*., did not change for three successive time points. We found that in addition to a wide spectrum of sporulation efficiencies, these strains also showed a specific pattern in the kinetics of sporulation, with the high sporulation efficiency strains showing fast sporulation kinetics and the low sporulation efficiency strains showing slow sporulation kinetics. For example, the strain YPS128 had maximum sporulation efficiency of 99.5% and reached saturation within 48 h. On the other hand DBVPG1788 had a maximum sporulation efficiency of 41.0% and took 8 days to reach this efficiency; keeping the strain any further in the sporulation condition did not increase the sporulation efficiency (Figure 1, Table S3). A comparison of sporulation efficiency estimated at 23°C [11] with our estimates (at 30°C) showed notable differences (see Table 1). However, there were 16 strains that were consistent in showing either high or low sporulation efficiency in both the datasets, which indicated that individual strains differed widely in the extent to which their sporulation efficiencies were temperature dependent.

### SNP Variation in Sporulation Genes

Since our sample size was limited, we chose to use only known sporulation and sporulation associated genes for our association analysis. A survey of the literature gave us a comprehensive list of 397 genes [3]–[5], [16] which included genes required for metabolic adaptation, early, middle and late sporulation genes (meiosis, spore formation and general stress response genes), mitochondrial and autophagy genes and also genes which were induced upon sporulation but had unknown function (Table S1). We then looked for variation in these genes across all strains by obtaining variant alleles from the SGRP alignment of all 32 strains. In total, we found 42,003 SNPs across these genes. The presence of variation allowed us to look for genetic determinants of variation of sporulation efficiency in these strains.

### Association Mapping of Sporulation Efficiency

We used two methods to identify SNPs in genes that were associated with an increase or decrease in sporulation efficiency. The first method used the LOD score to identify SNPs in which the genotype was strongly associated with the sporulation efficiency phenotype. A high LOD score was evidence for the presence of a quantitative trait locus, where the two genotypes at a locus had significantly different phenotype averages. The second method binned the strains into three classes of high, intermediate and low sporulation efficiency and then applied a binomial test (see method section) to identify SNPs in association with high and low sporulation. An overlap of both the methods revealed a list of 31 SNPs in 24 different genes (Bonferroni corrected p-value <0.03, permutation test) to be associated with sporulation efficiency variation (Table 2, Table S4).

### Population Structure Correction

Recently, the SGRP collection has been proposed for use in yeast GWAS studies [17], [18]. However, several issues have been raised about using this collection, including high type I errors (false positives) in determining causative loci [17], as underlying population structure can lead to spurious associations [18]. Using STRUCTURE [19] to determine population structure, and data for 201 phenotypes (not including sporulation efficiency), Diao and Chen [18], used extensive simulations and several GWAS methods on a genome wide set of tag SNPs to show that the mixed linear model EMMAX-KLA (a model with local ancestry and the kinship matrix as covariates) was the most effective at reducing type I errors and correcting for population structure in these strains. EMMAX-KLA was applied to our phenotype data to identify the tag SNPs that were significantly associated with the sporulation phenotype after correcting for population structure (p<0.05). We verified that the SNPs that we identified as statistically significant using the LOD score and binomial test were in perfect linkage disequilibrium (r^2^ = 1) with the tag SNPs identified as statistically significant using EMMAX-KLA, demonstrating that the association with sporulation efficiency remained after correcting for population structure (details in Table S4).

### Candidate SNPs and Genes Associated with Sporulation Efficiency

The SNPs that were identified as statistically significant by our two association analyses fell into two linkage blocks, one with a LOD score of 4.47 (Bonferroni corrected p<0.004, permutation test) and another with a LOD score of 3.5 (Bonferroni corrected p<0.026, permutation test). The first linkage block contained 5 SNPs whereas the second linkage block contained 26 different SNPs. The main result of our study was that these blocks of linked SNPs contained SNPs that were associated with sporulation efficiency in the SGRP strains. The SNPs in these clusters showed perfect linkage (r2=1) *i.e.* they segregated in an identical manner across the yeast strains and they were not all contiguous in the genome. Such a scenario could be possible due to population structure or a small sample size. Due to the occurrence of these linkage blocks, we could not computationally determine which of the SNPs in our clusters were causally associated with the phenotype and which were non-causal and linked to causal variants. Analysis of additional strains or additional experiments on the SGRP strains will be necessary to answer this question.

In order to identify candidate functional SNPs (*i.e.* causal variants) within these linked clusters, we looked up the gene annotations for these SNPs, as well as whether they were regulatory, synonymous or non-synonymous substitutions. Analysis of the sequence of these 24 genes revealed that 20 genes had SNPs in the coding region and 5 genes (CDC10, EMI5, MLS1, SPR6 and SSN8) had SNPs in the un-translated region, with SPR6 showing association both in coding and regulatory regions (see Table S4). Interestingly, deletions of EMI5, MLS1 and SSN8 have been reported to decrease sporulation efficiency [20] and CDC10 deletion abrogates sporulation [2].

Four of 26 coding SNPs were non-synonymous and could therefore affect the sporulation efficiency of a strain by altering binding ability, the extent of functionality or the flux through pathway and protein levels. Two of the 4 non-synonymous substitutions were in *SET3(A1783T)*, a repressor of sporulation specific genes [21] and *HOS4(A1384G)*, a component of Set3 complex and a suppressor of early and middle sporulation specific genes [22]. A possible reduction in protein function due to these mutations in the repressors, Set3 and Hos4, could lead to an increase in sporulation efficiency in strains with these SNPs. The other two non-synonymous substitutions were *MCK1(C1112A)* and *SPO74(C16A)*, deletions of which lead to decrease [2] and absence [23] of sporulation respectively. Among these four non-synonymous substitutions, the only one non-conservative substitution in Mck1(T371K) lies within its putative kinase domain, a positive regulator of meiosis and spore formation [24].

Two of the genes, *HOS4* and *SPR6* (a gene of unknown function expressed during sporulation and interacting with sporulation genes [25]), were present in both significant clusters (Table 2), suggesting their role as potential candidates for variation in sporulation efficiency across SGRP strains. However, an experimental validation is required to confirm their actual role, either by performing reciprocal hemizygosity analysis [26] or by constructing allele replacement strains.

## Discussion

The limited understanding of traits in natural populations is one of the biggest challenges in genetic association studies. The lack of information about phenotypes in the wild has limited our knowledge about the role of evolution, life history, environment and selection pressure in driving these processes. In this study, we have tried to understand genetic basis of variation in sporulation efficiency in natural isolates of yeast using the SGRP collection. Since sporulation is triggered as a response to nutrient deprivation, we predicted that the genetic factors contributing to variation in sporulation efficiency might be different between experimental populations and natural isolates. We measured sporulation efficiency of *S. cerevisiae* strains in the SGRP collection and found a large variation in sporulation efficiencies ranging from 0% to 100%, which could thus be used for dissecting the genetic basis of variation in the wild yeast strains.

We found both regulatory and coding variants responsible for variation in sporulation efficiency. Interestingly, only 15% (4/26) of coding variants were found to be non-synonymous mutations in *HOS4*, *MCK1*, *SET3* and *SPO74* which indicated that these genes could be the primary drivers of variation in sporulation efficiency in SGRP collection. Previous studies have identified roles for sporulation genes (*IME1*, *RME1*) and sporulation-associated genes (*FKH2*, *PMS1*, *RAS2*, *RSF1*, *SWS2*), as well as non-sporulation pathway genes (*MKT1*, *TAO3*) in maintaining this variation [6]–[8]. Our results showed that in the SGRP collection, a different set of genetic factors contribute to variation in sporulation efficiency.

*S. cerevisiae* is a powerful system for quantitative trait genetics and has advanced our understanding of the genotype-phenotype relationship of these traits. With decreasing cost of sequencing and high-throughput phenotyping, yeast can become a model for GWAS studies [17], [18]. Our results provide another example of how GWAS studies in yeast SGRP collection can identify known and new candidates for sporulation efficiency variation in natural strains. Thus, it provides an insight into how the selection pressure due to changes in the environmental conditions of natural isolates (such as nutrient availability) can drive evolution of a phenotype (such as variation in sporulation efficiency).

## Supporting Information

Table S1 List of 397 sporulation and sporulation associated genes used in this study (from refs. [3]–[5], [16]).
(XLSX)

Table S2 List of top 69 SNPs identified by the LOD score cutoff of 2.50 and validated by the binomial analysis.
(XLSX)

Table S3 Sporulation efficiency kinetics data for all *S. cerevisiae* strains in SGRP collection.
(XLSX)

Table S4 List of all significant SNPs with genome coordinates, their LOD score (LOD >3.50 and Bonferroni corrected p-value <0.03) and corresponding synonymous, non-synonymous or regulatory SNP changes. All of these SNPs were also not associated with population structure using EMMAX-KLA at 95% confidence (from Diao and Chen [18]).
(XLSX)
